# Shining Light on Protein Kinase Biomarkers with Fluorescent Peptide Biosensors

**DOI:** 10.3390/life12040516

**Published:** 2022-03-31

**Authors:** May C. Morris

**Affiliations:** Institut des Biomolécules Max Mousseron UMR 5247, Pôle Chimie Balard Recherche, Université de Montpellier, 1919 Route de Mende, 34293 Montpellier, France; may.morris@umontpellier.fr

**Keywords:** fluorescent biosensor, peptide, kinase activity, cancer

## Abstract

Protein kinases (PKs) are established gameplayers in biological signalling pathways, and a large body of evidence points to their dysregulation in diseases, in particular cancer, where rewiring of PK networks occurs frequently. Fluorescent biosensors constitute attractive tools for probing biomolecules and monitoring dynamic processes in complex samples. A wide variety of genetically encoded and synthetic biosensors have been tailored to report on PK activities over the last decade, enabling interrogation of their function and insight into their behaviour in physiopathological settings. These optical tools can further be used to highlight enzymatic alterations associated with the disease, thereby providing precious functional information which cannot be obtained through conventional genetic, transcriptomic or proteomic approaches. This review focuses on fluorescent peptide biosensors, recent developments and strategies that make them attractive tools to profile PK activities for biomedical and diagnostic purposes, as well as insights into the challenges and opportunities brought by this unique toolbox of chemical probes.

## 1. Introduction—Protein Kinases: Disease Biomarkers and Therapeutic Targets

**Figure 1:**
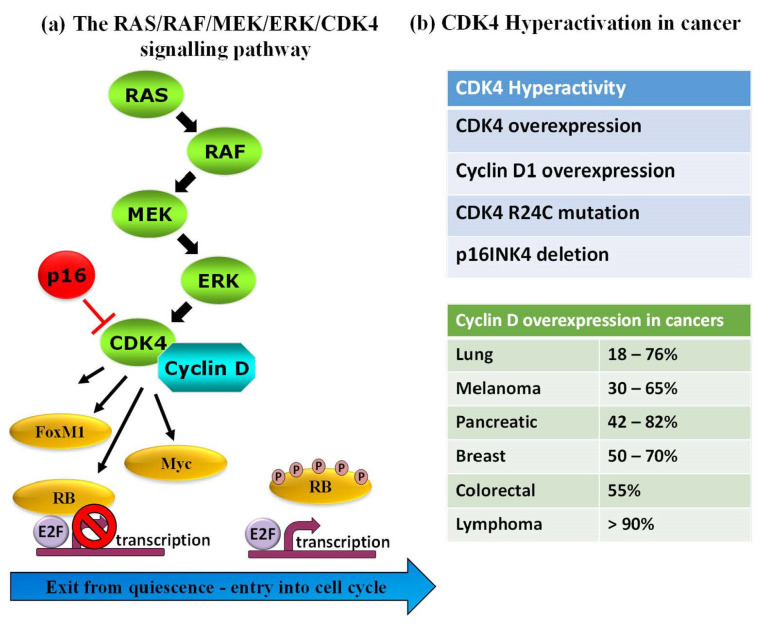
Complexity of the protein kinase activity/function illustrated by the RAS/RAF/MEK/ERK/CDK4 pathway. (**a**) PK activity is the result of a number of factors: CDK4 expression level, its regulation by activating and inhibitory partners, such as cyclin D and structural inhibitor p16INK4, posttranslational modifications catalysed by upstream regulators, such as ERK, MEK, RAS and RAF. (**b**) In human cancers, CDK4 hyperactivity may occur through different mechanisms that affect CDK4, cyclin D or p16INK4.

Protein kinases (PKs) are important and established gameplayers in a wide variety of biological processes, and their roles in various signalling pathways are now well-documented. PKs are also notorious for their dysregulation in human diseases, and several genetic, transcriptomic and proteomic studies report on rewiring of kinase networks in different pathologies, including cancer [1,2,3,4,5,6]. A large body of evidence highlights PK dysregulation in human cancers, in particular PKs involved in cell cycle progression, cell proliferation and checkpoint signalling [7,8,9,10,11]. As such, PKs constitute attractive pharmacological targets, and an arsenal of FDA-approved inhibitors is now available for therapeutic purposes and use in the clinic for different types of cancer [7,8,9,10,11,12,13,14,15,16,17]. For example, B-Raf is targeted by dabrafenib and vemurafenib, MEK1/2—by trametinib (widely used in melanomas), CDK4—by abemaciclib, palbociclib and ribociclib in breast cancer, Bcr-Abl are targeted by dasatinib and imatinib in CML, erlotinib and gefitinib target EGFR in lung cancers (NSCLC). Despite the central role of PKs in physiopathological pathways, the validation of several PKs as established relevant disease biomarkers and the availability of FDA-approved PK inhibitors for clinical treatment, few approaches enable direct detection of functional alterations of these enzymes in pathological samples for diagnostic purposes. Indeed, although the relative abundance of PKs can be readily determined through standard antigenic approaches such as Western blotting or ELISA, there is not always a direct correlation between PK levels and their functional activity, the latter being regulated by a network of upstream regulators, interacting partners and posttranslational modifications that affect their subcellular localization, conformation and overall function [18,19]. For instance, CDK4, a cyclin-dependent kinase that coordinates entry into the cell cycle and progression through the G1 phase lies downstream of the RAS/RAF/MEK/ERK pathway where it becomes activated upon binding to its cyclin D partner to form an active complex, but it is also held in check by structural inhibitor p16INK4 (cyclin-dependent kinase inhibitor 2A). CDK4 expression levels may not vary in different cell lines or patients, but may be subject to the R24C mutation (which prevents the p16INK4 binding), or p16INK4 may be deleted, or cyclin D may be overexpressed, all of which lead to kinase hyperactivation in melanoma and other cancers [20,21,22,23,24] (Figure 1).

## 2. Detection and Characterization of Protein Kinase Expression and Function

**Figure 2:**
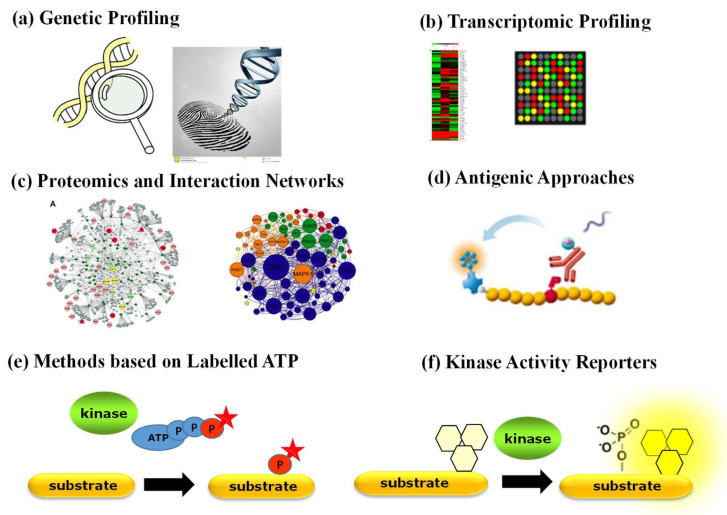
Strategies to detect and quantify PK expression and function: (**a**) genetic profiling; (**b**) transcriptomic profiling; (**c**) proteomics and interaction networks based on mass spectrometry analyses; (**d**) antibody-based approaches (ELISA and Western blotting); (**e**) methods based on ATP labelling; (**f**) fluorescent biosensors—kinase activity reporters.

Aside from antibody-based techniques that merely enable quantification of PK expression, a variety of other strategies have been implemented to characterize the status of PKs in physiopathological contexts (Figure 2). Genetic profiling of PKs proves useful when a PK of interest bears a specific mutation, undergoes genetic amplification or chromosomal rearrangement. Proteomic approaches based on mass spectrometry provide a wealth of quantitative information corresponding to a snapshot of phosphorylated substrates [3,4]. Several comprehensive studies report on the identification of interacting networks between PKs and their substrates through mass spectrometry approaches yielding large phosphoproteomics datasets, which are complemented by biochemical studies of kinase–substrate relationships through protein microarrays and computational prediction methods and further implemented into bioinformatic platforms that compile biochemical and structural information with protein substrate/partner networks, such as KinMap, RoKAI, PhosphoPredict, KinasePhos, KinaseXplorer and NetPhorest [3,4,25,26,27,28,29,30]. Although large-scale proteomic studies have yielded a wealth of “omics” big data, they provide insight into interaction networks based on the identification of substrate phosphorylation sites, which indirectly point to candidate kinase activities, but they neither report directly on functional activity of PKs nor constitute tools for point-of-care diagnostics. Moreover, neither of these approaches reports on the dynamic behaviour or kinetics of PK activities.

Several kinase activity assays relying on radioactive, fluorescent or antigenic components are commonly used in vitro with purified kinases [31,32] but remain limited for quantification of PK activities in complex samples, such as cell extracts, tissue or tumour biopsies. Indeed, the most common currently implemented approaches to study PK activity ex vivo rely on radioactive or fluorescently labelled ATP based either on incorporation of a detectable phosphate group onto artificial substrates by a specific kinase in vitro or on antigenic approaches, which depend on highly specific antibodies to recognize the phosphorylated form of the kinase substrate(s). Although these assays have been widely used in the laboratory, they remain indirect, require prior purification of the kinase of interest and multiple reagents and are somewhat time-consuming.

The lack of direct and standardized approaches to quantify functional PK activities in complex pathological samples (such as tumour biopsies or suspect lesions) makes it difficult to assess whether these enzymes are indeed dysregulated biomarkers and whether they may constitute relevant targets for therapeutic intervention. Profiling PK activities that are dysregulated in disorders or involved in pathogenesis and, therefore, constitute molecular disease biomarkers could provide a solid basis for the development of molecular diagnostics. Tools tailored to profile PK activity and identify enzymatic alterations associated with disease would provide precious functional information which cannot be obtained by genetic, transcriptomic or proteomic approaches.

## 3. Fluorescent Biosensors: Tools to Probe PK Activity in a Complex Yet Natural Physiopathological Environment

Detection of PK activities in complex environments such as the cellular or tissular milieu is a highly challenging task. Indeed, each PK is surrounded by a myriad of other proteins that may limit access and/or interfere with its detection. Moreover, the cellular concentration of most PKs is very low, in the pico–nanomolar range. More generally, detecting and tracking biomolecules in living cells and organisms and further attempting to monitor changes in its spatiotemporal dynamics, relative abundance and biological activity in response to specific stimuli or treatment with drugs is an extremely challenging task which amounts to finding a needle in a haystack. Overcoming this challenge requires the design of highly selective and sensitive probes, fine-tuned biosensing technologies to monitor the behaviour of one’s favourite biomolecule within its native physiological environment with high spatial and temporal resolution whilst accounting for the overall dynamic complexity of the system. Biosensors constitute potent tools for probing biomolecules in their natural environment and identifying disease biomarkers within complex samples such as plasma, urine and cell extracts since they can be tailored to recognize their target with exquisite selectivity and sensitivity [33,34,35]. Not only can fluorescent biosensors illuminate targets of interest and report on their presence, they may be designed to report on dynamic changes in enzymatic activity or conformation through sensitive changes in fluorescence emission of one or several fluorescent probes [36,37,38].

**Figure 3:**
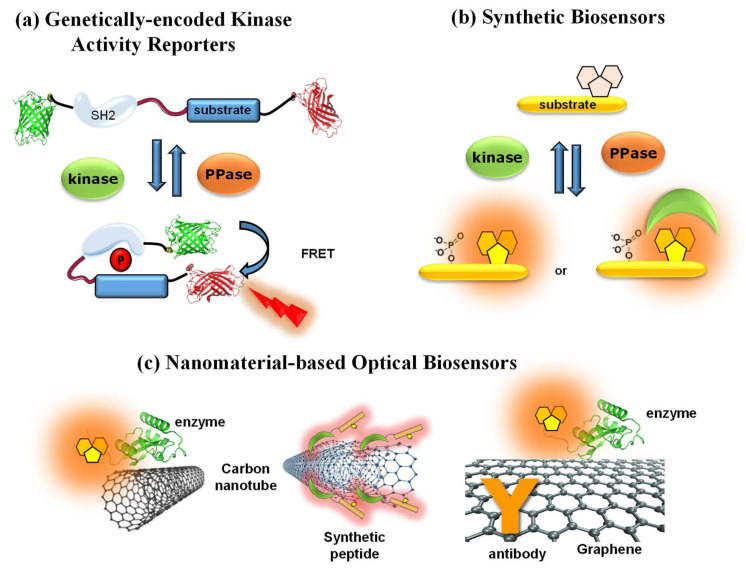
Toolbox and diversity of fluorescent biosensors. (**a**) Genetically encoded FRET kinase activity reporter; (**b**) synthetic peptide biosensors; (**c**) nanomaterial-based biosensors: single-wall and multiwall carbon nanotube biosensors (**left** and **middle**, respectively) and a graphene biosensor with antibodies and fluorescent enzymes (**right**).

Over the past decade, a wide variety of fluorescent biosensors have been developed to monitor and image protein kinase activities, providing means of gaining insight into kinase behaviour, and in some cases offering new technologies for high-throughput screening or diagnostic approaches, as described in several comprehensive reviews [38,39,40,41,42,43,44]. The Fluorescent Biosensor Toolbox comprises a highly diverse panel of fluorescent and luminescent biosensors which differ in their nature, including genetically encoded, synthetic, peptide-based and nanomaterial-based biosensors (Figure 3) [39,40,41,45,46,47,48,49,50,51,52,53,54,55].

Genetically encoded fluorescent biosensors have been being developed since the 1990s, initially prompted by the discovery and engineering of GFP into fusion proteins that could be expressed in living cells, which were tailored into FRET (fluorescence resonance energy transfer)-based biosensors, in particular reporters of kinase activities, also known as KARs (kinase activity reporters) [52,53,54,56,57,58]. Several genetically encoded FRET-based fluorescent biosensors have been developed to monitor aberrant kinase activity associated with cancer, such as Bcr-Abl biosensor Pickles implemented to measure hyperactivity of this kinase in cells from patients with chronic myelogenous leukaemia (CML) and evaluate kinase inhibitor efficacy, response to therapy and onset of resistance or the Src biosensor applied to study spatial control of Src in pancreatic cancer by means of intravital FLIM-FRET imaging [58,59,60,61,62,63,64]. However, these systems require transfection and time for ectopic expression in living cells and are therefore poorly suited for ex vivo diagnostic assays.

In contrast, nongenetic synthetic fluorescent reporters based on polymeric or polypeptide scaffolds conjugated to small organic fluorophores are better suited for ex vivo diagnostics as they provide controlled platforms that can be readily implemented to probe targets of interest within complex samples. A vast array of synthetic biosensors have been engineered to report on PK activities, including peptides derived from PK substrates, conjugated to solvatochromic probes, which respond either directly to proximal phosphorylation or indirectly following interactions with phosphorecognition domains that alter biosensor fluorescence or through quenching/unquenching strategies, as described below (reviewed in [39,40,41,42,43,44,45,46,47,48,49,50,51]). Synthetic biosensors offer attractive opportunities for molecular diagnostics, monitoring of disease progression and response to therapeutics, and several synthetic fluorescent biosensors have been developed for predictive purposes, for instance, to detect dysregulated PK biomarkers in leukaemia [65] or for point-of-care monitoring of therapeutic drugs in plasma and urine [66].

## 4. Design and Characterization of Fluorescent Peptide Biosensors

Peptides offer several major advantages inherent to their nature as they can be readily produced by means of synthetic chemistry, are easy to handle and store and can be labelled with synthetic fluorophores to yield fluorescent biosensors. They can be derived from substrates, docking sequences or complementary biomolecular recognition interfaces and constitute scaffolds and platforms for site-selective incorporation of fluorophores [67]. They can be further modified to optimize response and signal-to-noise ratio through introduction of quenchers or caging of specific amino acids to yield photoactivatable systems.

**Figure 4:**
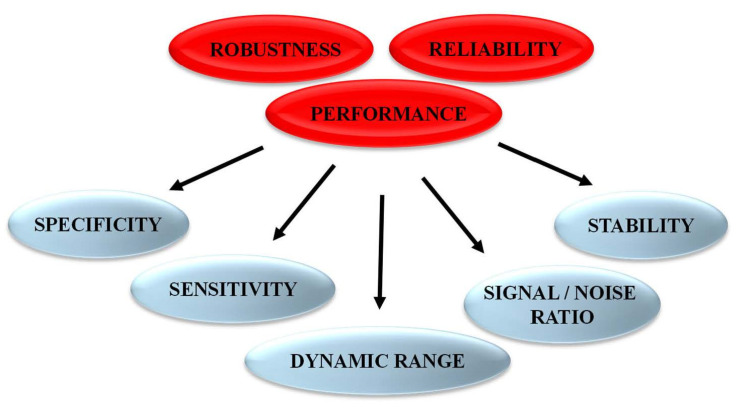
Features characterizing performance and robustness of fluorescent biosensors.

Following the rational design of fluorescent biosensors and their validation to recognize the target of interest and report on its activity, it is essential to characterize several features to achieve in-depth information and determine biosensor sensitivity and selectivity for the target/biomarker of interest over other targets. Biosensors should also be characterized to demonstrate their lack of (or insignificant) response to nonspecific environmental factors, including different solvents with different pH, polarity or viscosity, which might affect the photophysical properties of the fluorophore(s). Further optimization to improve biosensor performance, in particular the signal-to-noise ratio, the limit of detection, the linear range of response and the dynamic range constitute critical points to yield highly specific, sensitive and robust biosensors (Figure 4).

### 4.1. Incremental Complexity and Sensitivity of Fluorescent Peptide Biosensor Designs

Fluorescent peptide reporters of PK activity are engineered through a subtle combination of peptide substrate sequences with synthetic fluorophores selected to best respond to the needs of the application. Careful fluorophore selection and positioning within the peptide scaffold are critical to optimize and achieve the maximal response associated with kinase activity. Several strategies have been developed to design biosensors that respond to kinase activity through sensitive changes in fluorescence emission thanks to incorporation of environmentally sensitive fluorophores that respond to changes in polarity in their local environment [67,68,69,70].

**Figure 5:**
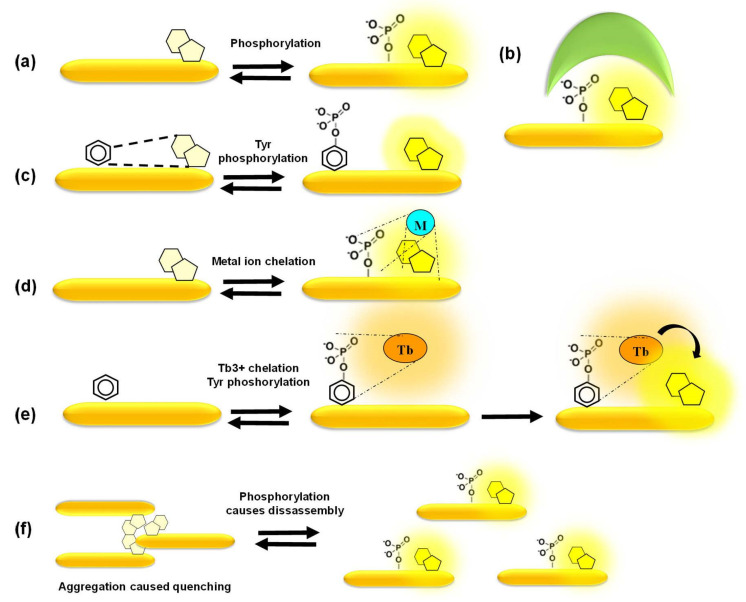
Mechanisms of response of fluorescent peptide biosensors. (**a**) Phosphorylation of the peptide affects fluorescence emission of the fluorophore proximal to the phosphate group; (**b**) phosphorylation of the peptide substrate promotes binding of a phosphoamino acid-binding domain which alters the local environment; (**c**) a quencher proximal to the fluorophore reduces its basal fluorescence until phosphorylation disrupts quenching; (**d**) chelation-enhanced fluorescence of a fluorophore upon binding of a metal ion involved in the phosphorylation process; (**e**,**f**) aggregation-caused quenching of fluorophores is reversed upon phosphorylation which promotes disassembly of fluorescent biosensors, leading to fluorescence enhancement.

In the simplest scenario, phosphorylation itself, at a position proximal to the fluorophore, is sufficient to promote changes in fluorescence emission (Figure 5a). In a more complex design, a phosphoamino acid-binding domain (PAABD) is included, which binds the phosphorylated peptide substrate, thereby significantly altering the local environment of the fluorophore (Figure 5b). The signal-to-noise ratio may be even further improved through quenching of basal fluorescence by a neighbouring amino acid or a synthetic quencher whose interaction with the fluorophore is disrupted upon phosphorylation of the peptide substrate by the kinase of interest and/or by the additional binding of a PAABD (Figure 5c). David Lawrence’s group pioneered these strategies to develop some of the first tyrosine biosensors that are quenched by pyrene, that he dubbed “self-reporting biosensors”, and further made use of the exogenous SH2 and 14–3–3 domains as PAABDs to develop more sensitive biosensors for tyrosine and serine/threonine PKs, respectively. He further designed the “deep quench” strategy to enhance the fluorescence response of cAMP-dependent PK biosensors, in which phosphorylation of the peptide substrate disrupts interactions between the fluorophore and a quencher and further promotes binding of the abovementioned PAABDs [71,72,73].

Alternatively, highly selective mechanisms of fluorescence enhancement have been designed, such as chelation-enhanced fluorescence of a fluorophore upon binding of a metal ion involved in the phosphorylation process or aggregation-caused quenching reversed by phosphorylation-induced dissociation [39,40,41,65,66,67,68,69,70] (Figure 5d–f). Barbara Imperiali’s group developed a smart and sensitive strategy based on sulfonamido-oxine (Sox)-conjugated peptides, in which the Sox dye binds Mg^2+^ upon phosphorylation of the substrate peptide, thereby undergoing chelation-enhanced fluorescence. Her group further designed a “recognition domain-focused” strategy involving introduction of the Sox-modified cysteine and incorporation of extended binding determinants to improve selective recognition by the kinase of interest, thereby developing highly sensitive and selective peptide biosensors for serine/threonine PKs (PKCs, Pim2, Akt1, MK2 and PKA) and tyrosine PKs (IRK, Src, Abl). Moreover, a docking domain strategy was devised in order to engineer highly selective ERK1/2 biosensors by including a domain derived from the Ets-1 substrate of these kinases that facilitates its recognition and binding affinity to ERK1/2 compared to the peptide substrate alone. Sox-based biosensors have been further implemented to develop a multiplexed fluorescence-based assay for PK activities in cell lysates [74,75,76,77]. Laurie Parker’s group engineered several biosensors based on chelation of Tb3+. Time-resolved luminescence of these biosensors upon terbium sensitization reports on phosphorylation by tyrosine kinases such as Syk, ALK and Abl, leading to the development of the KINATEST-ID pipeline for terbium-based tyrosine kinase assays. This group further took advantage of terbium luminescence to excite organic fluorophores conjugated to phosphotyrosine biosensor peptides through energy transfer, thereby generating orthogonal biosensors for Lyn and Syk within the same assay. This group also developed a set of cell-penetrating derivatives of the Abl and Src family biosensors conjugated with organic fluorophores by including a TAT sequence to generate FLIM probes for imaging purposes, whose fluorescence lifetime increases upon tyrosine phosphorylation [78,79,80,81].

The currently available biosensor designs range from peptide substrates onto which environmentally sensitive dyes are conjugated for sensitive and selective response to the PKs of interest to more elaborate designs incorporating solvatochromic dyes, lanthanides or fluorophores whose activation is dependent on smart and selective mechanisms, thereby yielding tools with an extremely high signal-to-noise ratio that can essentially switch ON/OFF in response to phosphorylation. Although the vast majority of these fluorescent biosensors are essentially used in vitro, they are being increasingly implemented for imaging purposes thanks to covalent or noncovalent facilitated delivery strategies in combination with cell-penetrating peptides [82,83,84].

### 4.2. Toolbox of Cyclin-Dependent Kinase Peptide Biosensors

Cyclin-dependent kinases (CDK/cyclins) form a family of heterodimeric Ser/Thr protein kinases that rely on binding of a cyclin or a cyclin-like partner for activation of the CDK subunit. Initially identified for their key roles in coordination of cell cycle progression, cell growth and division, they were later found to participate in several essential biological processes including metabolism, transcription haematopoiesis, angiogenesis and neuronal differentiation [85,86,87,88,89,90,91,92]. These kinases are frequently hyperactivated in human cancer and constitute established biomarkers and attractive pharmacological targets for anticancer therapeutics [8,9,10]. Their hyperactivity can result from one of many mechanisms, including genetic amplification, protein overexpression, expression of truncated forms of the cyclin partner, as frequently observed for cyclin E, or point mutations that confer constitutive activity, such as CDK4 R24C which prevents inhibition by endogenous inhibitor p16INK4. Moreover, several CDKs appear to have redundant or partially overlapping functions and substrates in common. Hence, probing and quantifying the hyperactivity of these kinases in their native environment remains challenging.

**Figure 6:**
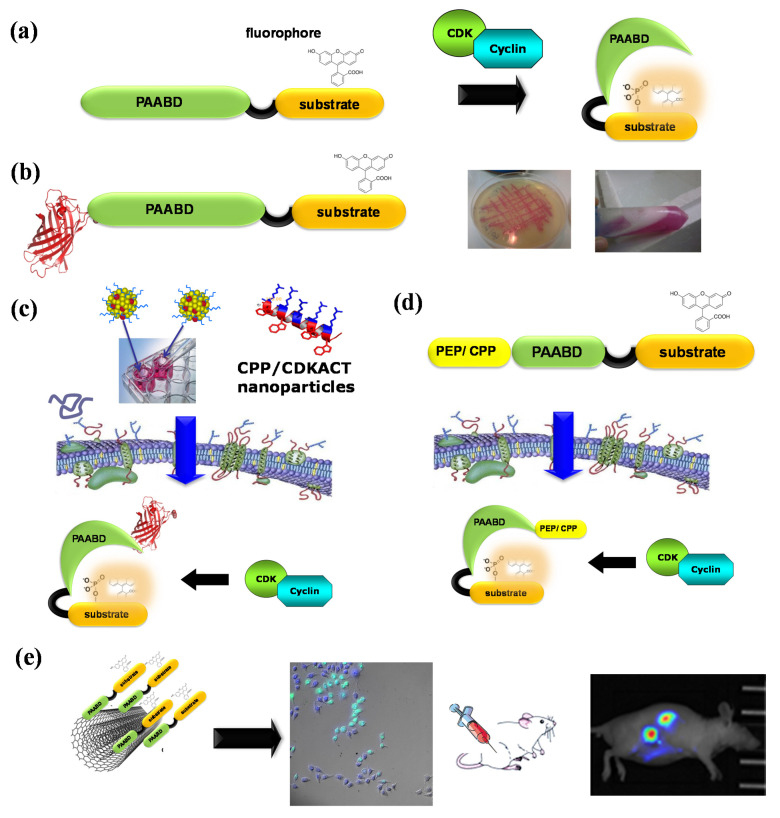
CDKACT fluorescent peptide biosensor technology. (**a**) Schematic representation of CDKACT biosensors: bipartite biosensors comprising a CDK-specific substrate moiety (orange) onto which an environmentally sensitive dye is conjugated, a short linker and a phosphoamino acid-binding domain (or PAABD, green) that folds onto the phosphorylated substrate, thereby altering the local environment of the fluorophore and promoting changes in fluorescence emission. (**b**) An RFP fusion of CDKACT expressed in *E. coli*. (**c**) CDKACTs or RFP-CDKACTs can be introduced into cultured cells for live imaging experiments through complexation with cell-penetrating peptides (Pep1) to form nanoparticles that cross cell membranes and release CDKACT into cells. (**d**) A self-cell-penetrating variant of CDKACT was generated through fusion of the N-terminal moiety of Pep1 to the PAABD of CDKACT5. (**e**) CDKACT1-multiwall carbon nanotube conjugates yield an ultrasensitive nano-biosensor for imaging the CDK1 activity in mice.

With the aim of developing original technologies for diagnostic applications, we have designed, engineered and established proofs of concept for fluorescent biosensor technologies to probe CDK/cyclins in vitro and in living cells: first, CDKSENS, a biligand peptide biosensor that reports on their relative abundance [93]; then, CDKACT, a family of polypeptide-based biosensors that report on the relative activity of CDK/cyclins with applications in vitro, in cultured cells and in vivo [94,95,96,97,98,99,100] (Figure 6). CDKACTs are bipartite biosensors that comprise a substrate moiety phosphorylated by a CDK, which is labelled with an environmentally sensitive dye and a PAABD that recognizes the phosphorylated substrate. Upon phosphorylation of the substrate moiety by a CDK, binding between the latter and the PAABD is strongly favoured, thereby modifying the local environment of the fluorophore, which leads to sensitive changes in its fluorescence emission. CDKACT biosensors were first engineered as recombinant proteins expressed in *E. coli*, purified by FPLC and labelled on a unique cysteine within the substrate moiety. An RFP fusion of a generic CDKACT derived from histone H1 was further engineered and complexed with cell-penetrating peptide Pep1 to promote its cellular internalization and measure CDK activities in cultured cells by means of fluorescence microscopy and time-lapse imaging associated with ratiometric quantification of the fluorophore relative to RFP fluorescence [94]. The second generation of CDKACT biosensors were peptides with a much shorter PAABD derived from the WW domain of Pin1, tailored to respond to CDK4, CDK5, CDK6 or CDK1 by replacing the histone H1 substrate moiety by sequences specifically phosphorylated by each of these kinases [95,96,97,98,99]. These modular synthetic fluorescent peptides report on the specific activity of cyclin-dependent kinases in a sensitive, direct and quantitative fashion through changes in fluorescence emission. Moreover, they can be introduced into living cells by complexation with cell-penetrating peptides (CPPs) for live cell imaging purposes. Alternatively, self-cell-penetrating variants can be engineered, as exemplified for CDKACT5, which bears a CPP sequence derived from Pep1 at the N-terminus of the PAABD, thereby enabling quantification of the CDK5 activity in U87 cells by means of fluorescence microscopy [97]. More recently, peptide biosensor CDKACT1 was immobilized onto multiwall carbon nanotubes, which serve both as platforms and carriers, thereby generating a nano-biosensor that readily penetrates cultured cells and enables in vivo imaging in mouse models [99].

**Figure 7:**
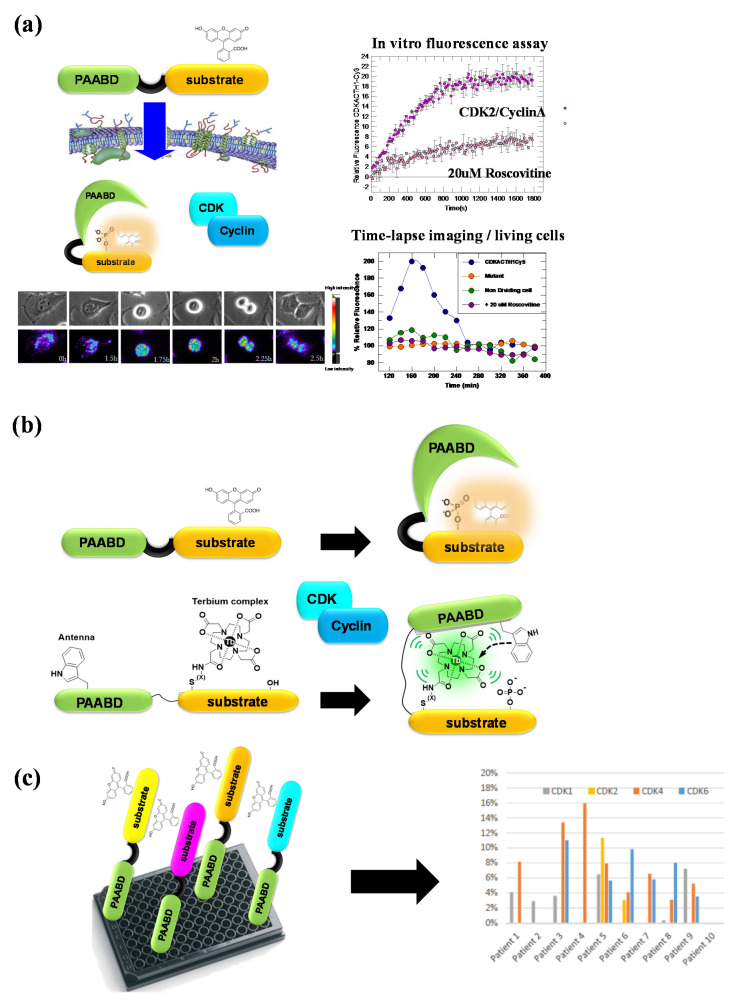
CDKACT fluorescent peptide biosensor technology. (**a**) CDKACT technology for quantifying the CDK activity in vitro by means of fluorescence spectroscopy and in living cells by means of fluorescence microscopy and live cell imaging following facilitated delivery into cultured cells. (**b**) Comparison of fluorescent and luminescent CDKACT4 biosensors. (**c**) Schematic representation of multiplex biosensing experiments performed by combining four different CDKACT biosensors to profile CDK1, CDK2, CDK4 and CDK6 in human cancer biopsies.

CDK-specific biosensors engineered to provide means of reporting on individual CDK activities also constitute a toolbox for multiplex detection of these kinases in healthy and cancer cell extracts and biopsies (Figure 7). CDKACT biosensors have more recently been combined to monitor several different CDKs in the same sample. For instance, we investigated the implication of CDKs in skin samples from patients with psoriasis and showed that CDK2 was upregulated, whereas CDK4 was inhibited by p16INK4 [100]. Using a TAMRA-labelled and a luminescent CDKACT4, we have investigated hyperactivation of CDK4 in melanoma cell lines and xenografts in mouse models treated with different CDK inhibitors [95,96]. We have further compared CDK4 and CDK6 activities in cell lines derived from patients with mesothelioma [98]. We have characterized the relative activities of different CDKs in a panel of cell lines derived from different cancers including lung cancer, breast cancer, melanoma and glioblastoma. More recently, we have undertaken multiplex profiling studies of CDK activities in lysates prepared from human biopsies of lung adenocarcinoma and lymphoma in collaboration with the Hospital of Montpellier (CRB-CHU Montpellier).

## 5. Concluding Remarks and Perspectives

Fluorescent biosensors constitute attractive tools to probe the function, regulation and spatiotemporal dynamics of protein kinases in fundamental studies, investigate complex signalling cascades that lead to kinase activation in the physiological context and report on their function in the cellular context. They can be implemented to monitor biomarkers in complex biological samples, such as cell extracts and living cells. In particular, fluorescent peptide biosensors constitute versatile and controllable platforms for target detection in vitro, as well as in living cells and in vivo by means of fluorescence imaging following facilitated delivery. In particular, a wide variety of peptide biosensors have been engineered to report on PK activities using different strategies to achieve optimal sensitivity and signal-to-noise response. These selective optical probes offer means of sensing functional alterations and quantifying differences in kinase activities between healthy and cancer cell lines and in response to PK inhibitors. As such, they constitute attractive alternatives to antibody-based approaches for detection of biomarkers in cancer diagnostics and can provide precious functional information.

These tools offer countless perspectives for biomedical applications in human disease and health monitoring, which are only limited by technical issues related to sensitivity, selectivity and robustness in complex samples. Tailoring fluorescent peptide biosensors to achieve the greatest selectivity and sensitivity involves rational design accounting for the application and the target’s context and incorporating the right fluorophore into the right peptide scaffold to achieve the most appropriate combination to match the question, target and environment. Design and engineering of FPBs for specific applications also entails accounting for bottlenecks associated with selective biomarker/target recognition and response, limit of detection, signal-to-noise ratio and reproducibility in complex samples.

Despite these limits, fluorescent peptide biosensors are indeed potentially amenable to predictive diagnostics, monitoring of disease progression and response to therapeutics thanks to their synthetic, controllable nature. They constitute powerful and promising tools for fighting cancer at an early stage, and we anticipate that they will enable widespread development of predictive diagnostics thanks to new generations of highly sensitive and solvatochromic fluorophores that can be conjugated onto peptide scaffolds with high specificity and selectivity for the PK biomarker of interest. Moreover, since most diseases are not monofactorial and involve a combination of molecular alterations, fluorescent biosensor technologies that can distinguish and report on a panel of relevant PK activities within the same sample offer cost- and time-effective means of profiling these biomarkers and identifying signatures associated with diseases.
